# Galectin-9 Ameliorates Clinical Severity of MRL/lpr Lupus-Prone Mice by Inducing Plasma Cell Apoptosis Independently of Tim-3

**DOI:** 10.1371/journal.pone.0060807

**Published:** 2013-04-09

**Authors:** Masahiro Moritoki, Takeshi Kadowaki, Toshiro Niki, Daisuke Nakano, Genichiro Soma, Hirohito Mori, Hideki Kobara, Tsutomu Masaki, Masakazu Kohno, Mitsuomi Hirashima

**Affiliations:** 1 Department of Cardiorenal and Cerebrovascular Medicine, Faculty of Medicine, Kagawa University, Kagawa, Japan; 2 Department of Immunology and Immunopathology, Faculty of Medicine, Kagawa University, Kagawa, Japan; 3 Department of Holistic Immunology, Kagawa University, Kagawa, Japan; 4 Department of Pharmacology, Faculty of Medicine, Kagawa University, Kagawa, Japan; 5 Department of Gastroenterology and Neurology, Faculty of Medicine, Kagawa University, Kagawa, Japan; Institut Jacques Monod, France

## Abstract

Galectin-9 ameliorates various murine autoimmune disease models by regulating T cells and macrophages, although it is not known what role it may have in B cells. The present experiment shows that galectin-9 ameliorates a variety of clinical symptoms, such as proteinuria, arthritis, and hematocrit in MRL/lpr lupus-prone mice. As previously reported, galectin-9 reduces the frequency of Th1, Th17, and activated CD8^+^ T cells. Although anti-dsDNA antibody was increased in MRL/lpr lupus-prone mice, galectin-9 suppressed anti-dsDNA antibody production, at least partly, by decreasing the number of plasma cells. Galectin-9 seemed to decrease the number of plasma cells by inducing plasma cell apoptosis, and not by suppressing BAFF production. Although about 20% of CD19^−/low^ CD138^+^ plasma cells expressed Tim-3 in MRL/lpr lupus-prone mice, Tim-3 may not be directly involved in the galectin-9-induced apoptosis, because anti-Tim-3 blocking antibody did not block galectin-9-induced apoptosis. This is the first report of plasma cell apoptosis being induced by galectin-9. Collectively, it is likely that galectin-9 attenuates the clinical severity of MRL lupus-prone mice by regulating T cell function and inducing plasma cell apoptosis.

## Introduction

Systemic lupus erythematosus (SLE) is a systemic autoimmune disease characterized by autoantibody production against self-antigens. Among SLE complications, lupus nephritis is the most serious and a major predictor of poor prognosis [1]. Until recently, glucocorticoids, aspirin and antimalarials were approved for treatment of SLE. B-cell stimulatory factors promote the loss of B-cell tolerance and drive autoantibody production. B cell activation mediated by B-cell activator factor belonging to the TNF family (BAFF) and a proliferation-inducing ligand (APRIL) have been implicated in SLE pathogenesis [2], [3], [4]. This suggests that B cell regulation, in addition to T cell regulation, is required for SLE treatment [2].

Gal-9 is a β-galactoside binding lectin that exhibits therapeutic effects in autoimmune disease models, such as autoimmune arthritis, experimental allergic encephalomyelitis, and Type 1 diabetes mellitus [5], [6], [7]. Such therapeutic effects of Gal-9 seem to be ascribed to the decrease of Th1 and Th17 effector cells expressing Tim-3 [8]. It has also been found that the decrease of Th1 and Th17 effector cells is likely induced by programmed cell death of effector cells through a Gal-9/Tim-3 interaction [8]. In contrast, Gal-9 expands Foxp3^+^ regulatory T cells (Tregs) in vivo and in vitro [5]. Furthermore, Gal-9 ameliorates immune complex (IC)-induced inflammation by suppressing IC-induced macrophage activation and C5a generation [9]. Collectively, Gal-9 seems to regulate a variety of immune cells to ameliorate autoimmune inflammation. Nevertheless, little is known about the effects of Gal-9 on B cell autoantibody production, although it is clear that B cells and B cell-derived autoantibody are associated with the pathogenesis of autoimmune disorders.

The purpose of the present study is to test whether Gal-9 ameliorates lupus signs and suppresses anti-dsDNA antibody production by inducing plasma cell apoptosis.

## Materials and Methods

### Mice

MRL/lpr lupus-prone and MRL/lpr^+/+^ mice were purchased from Japan SLC (Shizuoka, Japan). All mice were housed in plastic boxes in groups of 3 to 4 under a 12∶12 light cycle with food and water provided *ad libitum*.

The study protocol was approved by the Animal Care and Use Committee of Kagawa University, and mice used in this research received humane care to minimize suffering in accordance with international and national guidelines of humane laboratory animal care. Mice were sacrificed by CO_2_ narcosis unless otherwise specified.

### Experimental Protocol

All Gal-9 preparations used in the present experiment were >95% pure by SDS-PAGE with less than 0.001 endotoxin units/µg, as assessed by a limulus turbimetric kinetic assay using a Toxinometer ET-2000 (Wako, Osaka, Japan). Nine-week-old MRL/lpr lupus-prone mice were injected intraperitoneally with human stable Gal-9 with no linker peptide (30 µg/mouse, 3-times/week) or PBS as a control, to assess the therapeutic effects of Gal-9. Proteinuria, paw volume, and hematocrit were monitored until mice were 20 weeks of age.

Eight-week-old mice were treated with Gal-9 for 4 weeks to assess the effects of Gal-9 on the level of anti-dsDNA antibody and the frequency of splenic T and B cell subpopulations.

### Laboratory Methods

Proteinuria was measured using the BCA Protein Assay Reagent Kit (Takara Bio Inc., Otsu, Japan). Clinical signs of arthritis (i.e., paw swelling) were monitored during the course of disease by water displacement plethysmometry. Paw swelling was expressed as increased paw volume. Hematocrit values were collected from the tail vein (70 µl) in 1 mm heparinized tubes. The tubes were spun and hematocrit was determined using a Hawksley Micro-haematocrit reader (Lancing, Sussex, UK).

### Flow Cytometric Analysis

Spleen cells were obtained from PBS or Gal-9 treated MRL/lpr lupus-prone mice. Single-cell suspensions were prepared, and red blood cells removed using lysis buffer (BioLegend, San Diego, CA, USA). One million splenocytes were incubated for 30 min on ice in staining buffer with the relevant fluorochrome-labeled monoclonal antibodies. For intracellular cytokine and Foxp3 staining, the cells were fixed and permeabilized with Cytofix/Cytoperm solution (BD Biosciences, San Jose, CA, USA) and Foxp3 Fix/Perm Buffer Set (BioLegend) according to the manufacturer’s instructions. The following anti-mouse antibodies were used: IFNγ-FITC, CD4-PE, CD3-PerCP, Tim3-PE, (all from eBioscience, San Diego, CA, USA), CD138-PE (BD Biosciences), and Foxp3-Alexa488, IL-17A-PerCP, CD25-APC, CD8-Alexa488, CD44-APC, CD19-APC, NK1.1-PE, and GL-3-APC (all from BioLegend). All data were analyzed with a FACSCalibur flow cytometer (BD Biosciences) and Flowjo software (Tree Star, Ashland, OR, USA).

### Apoptosis

Plasma cells were purified from spleen in MRL/lpr lupus-prone mice using MACS CD138^+^ Plasma Cell Isolation Kit (Miltenyi Biotec) as recommended by the manufacturer. The cell population contained >98% CD138^+^ cells. The isolated plasma cells were used for apoptosis assay. The single cell suspensions were incubated for 5 h with 30 nM Gal-9 in 96 well flat-bottom plates in humidified incubators in the presence of 5% CO2. After the incubation period was over, the cells were stained for Annexin V (BioLegend) with or without 7AAD and analyzed immediately by flow cytometry.

We further assessed the effects of lactose (30 mM), sucrose (30 mM), Tim-3 mAb (10 µg/ml, eBioscience), and rat IgG2a (10 µg/ml, eBioscience) on Gal-9-induced apoptosis. All data were acquired with a FACSCalibur (BD Biosciences) and analyzed with FlowJo software (Tree Star).

### ELISA

The serum levels of anti-dsDNA antibody, total IgG and BAFF were measured with mouse anti-dsDNA antibody ELISA kit (Shibayagi, Gunma, Japan), mouse IgG ELISA kit (Immunology Consultants Laboratory, Inc., Portland, OR, USA) and mouse BAFF Immunoassay kit (R&D Systems, Minneapolis, MN, USA) according to the manufacturer’s instructions, respectively.

### Statistical Analysis

Student’s paired or unpaired t-tests and one- or two-way ANOVA were used for statistical comparisons. All statistical analyses were performed with Prism 5 software (Graphpad Software, La Jolla, CA).

## Results

### Galectin-9 Ameliorates Clinical Severity of MRL/lpr Lupus-prone Mice

**Figure 1:**
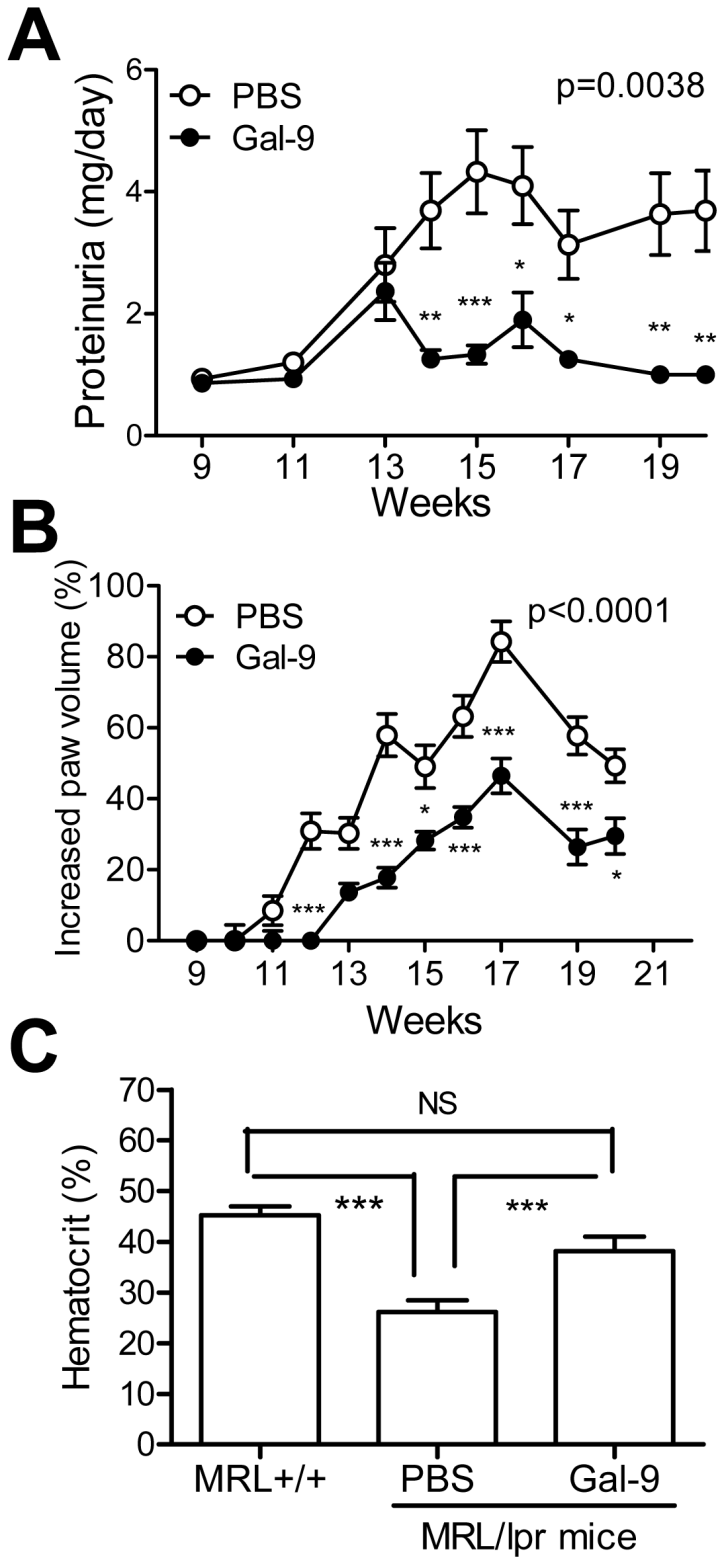
Effects of Gal-9 on lupus nephritis in MRL/lpr mice. (**A**) Comparison of proteinuria between PBS-treated (PBS) and Gal-9-treated (Gal-9) MRL/lpr lupus-prone mice. Gal-9 and PBS were injected intraperitoneally into 9-week-old MRL/lpr lupus-prone mice 3-times/week until they were 20 weeks of age. Mean and SEM of PBS treated mice (n = 10) and Gal-9 treated mice (n = 8) are shown. (*, P<0.05; **, P<0.01, ***, P<0.001). (**B**) Comparison of increase of paw volume between PBS and Gal-9-treated mice. Gal-9 or PBS was intraperitoneally injected to 9-week-old MRL/lpr lupus-prone mice 3-times/week until 20-weeks-old. Mean and SEM of PBS-treated (n = 10) and Gal-9-treated mice (n = 8) are shown. (*, P<0.05, ***, P<0.001). (**C**) Comparison of hematocrit between MRL^+/+^ (MRL/lpr^+/+^) mice and MRL/lpr (MRL/lpr^−/−^ lupus-prone) mice in 20-week-old mice following treatment with PBS or human Gal-9. (***, P<0.001).

We first evaluated the therapeutic effects of Gal-9 by treating 9-week-old MRL/lpr lupus-prone mice with Gal-9. Gal-9 significantly suppressed the level of proteinuria (**Figure 1A**). Since it is well known that MRL/lpr lupus-prone mice develop autoimmune arthritis and hemolytic anemia [11], [12], we also examined the effects of Gal-9 on these diseases. Gal-9 clearly retarded the onset of arthritis and suppressed the increase of paw volume (**Figure 1B**). Hematocrits of 20-week-old MRL/lpr lupus-prone mice were significantly lower than that of MRL/lpr^+/+^ mice. When MRL/lpr lupus-prone mice were treated with Gal-9, hematocrit increased significantly (**Figure 1C**). These results suggest that Gal-9 helps to ameliorate disease onset in MRL/lpr lupus-prone mice.

### Regulatory Effects of Gal-9 on T Cells

**Figure 2:**
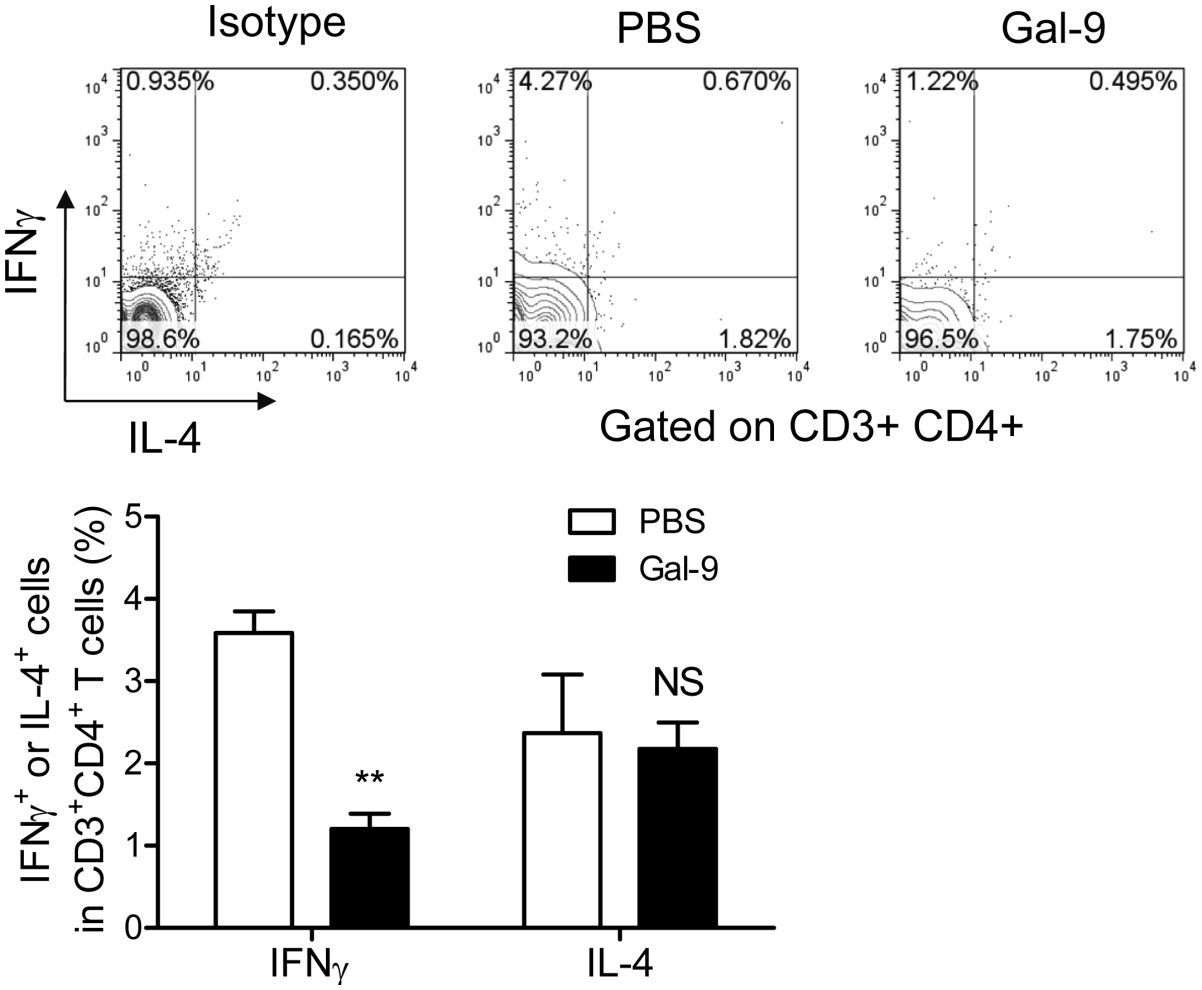
Effects of Gal-9 on splenic Th1 and Th2 subsets in MRL/lpr mice. Comparison of IFNγ^+^ Th1 and IL-4^+^ Th2 frequency between PBS-treated (n = 4) and Gal-9-treated (n = 4) mice (***, P<0.001). Representative data of flow cytometric profiles are shown.

It has been shown that Gal-9 induces apoptosis of Th1 and Th17 cells [6], [13], [14], [15]. Similarly, Gal-9 induces apoptosis of activated CD8^+^ T cells [16], [17]. Therefore, we treated 8-week-old MRL/lpr lupus-prone mice with Gal-9 to assess its effects on splenic effector T cells. MRL/lpr lupus-prone mice were sacrificed at 4 weeks after Gal-9 treatment (12-week-old). FACS analysis was first done to establish the frequency of splenic effector CD4 and CD8 T cells. We found that Gal-9 decreased the frequency of Th1 (IFNγ^+^ IL-4^−^ CD3^+^ CD4^+^ cells) significantly, while Th2 (IL-4^+^ IFNγ^−^ CD3^+^ CD4^+^ cells) were unchanged in Gal-9-treated MRL/lpr lupus-prone mice (**Figure 2**). In contrast, Gal-9 did not reduce γδT cells and NKT cells, and did not increase Foxp3^+^ CD4 T cells (**data not shown**).

**Figure 3:**
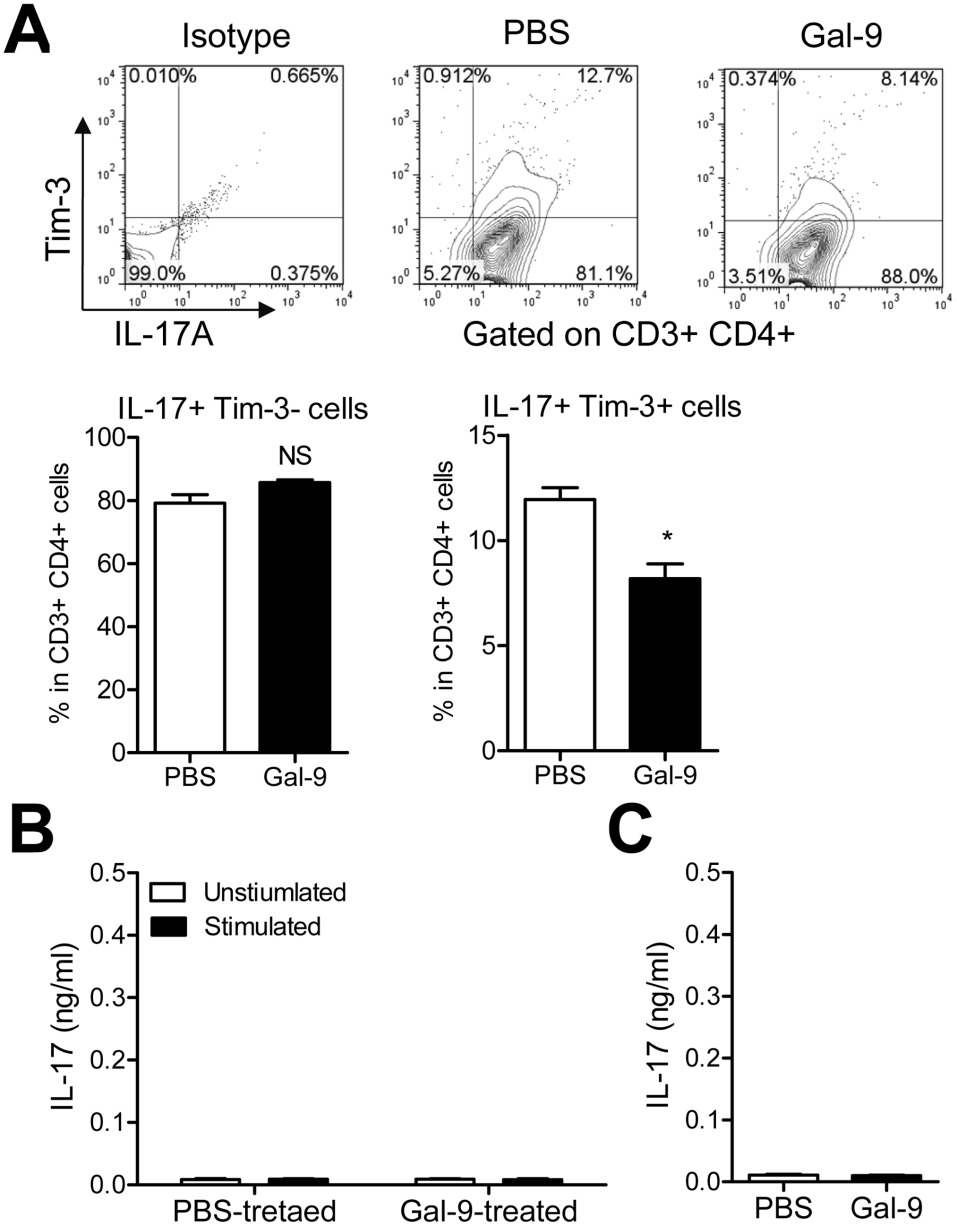
Gal-9 decreases splenic Th17 cells. (**A**) Comparison of frequency of Tim-3^+^ IL17A^+^ Th17 between PBS-treated (n = 4) and Gal-9-treated (n = 4) mice (**, P<0.01). Representative data of flow cytometric profiles are shown. (**B**) Spleen cells of MRL/lpr mice do not release IL-17. Spleen cells from PBS-treated (n = 4) and Gal-9-treated (n = 4) mice were cultured for 6 h with or without PMA+ionomycin. (**C**) Negligible level of IL-17 in plasma of MRL/lpr mice. PBS-treated (n = 4) and Gal-9-treated (n = 4) mice.

Surprisingly, most of CD4^+^ T cells expressed IL-17 in the cytoplasm, and there was no significant difference in the frequency of total IL-17^+^ Tim-3^−^ CD4 T cells between PBS- and Gal-9-treated MRL/lpr lupus-prone mice (**Figure 3A**). In contrast, the frequency of Tim-3^+^ IL-17^+^ CD4 T cells, probably Th17, was decreased by Gal-9 treatment similar to Th1.

We assessed ELISA assay to compare the level of IL-17 in the culture supernatants of spleen CD4 T cells from PBS- and Gal-9-treated mice with or without PMA+ionomycin stimulation. Surprisingly, the levels of IL-17 in the culture supernatants were negligible even after the stimulation, and there was no significant difference in IL-17 levels between PBS- and Gal-9-treated MRL/lpr lupus-prone mice, suggesting that release of IL-17 was impaired in most CD4 T cells of MRL/lpr lupus-prone mice (**Figure 3B**). We also assessed ELISA assay for IL-17 levels in plasma of PBS-treated and Gal-9-treated MRL/lpr lupus prone mice. Expectedly, ELISA assay revealed that plasma IL-17 level was negligible in both PBS- and Gal-9-treated MRL/lpr lupus-prone mice (**Figure 3C**).

**Figure 4:**
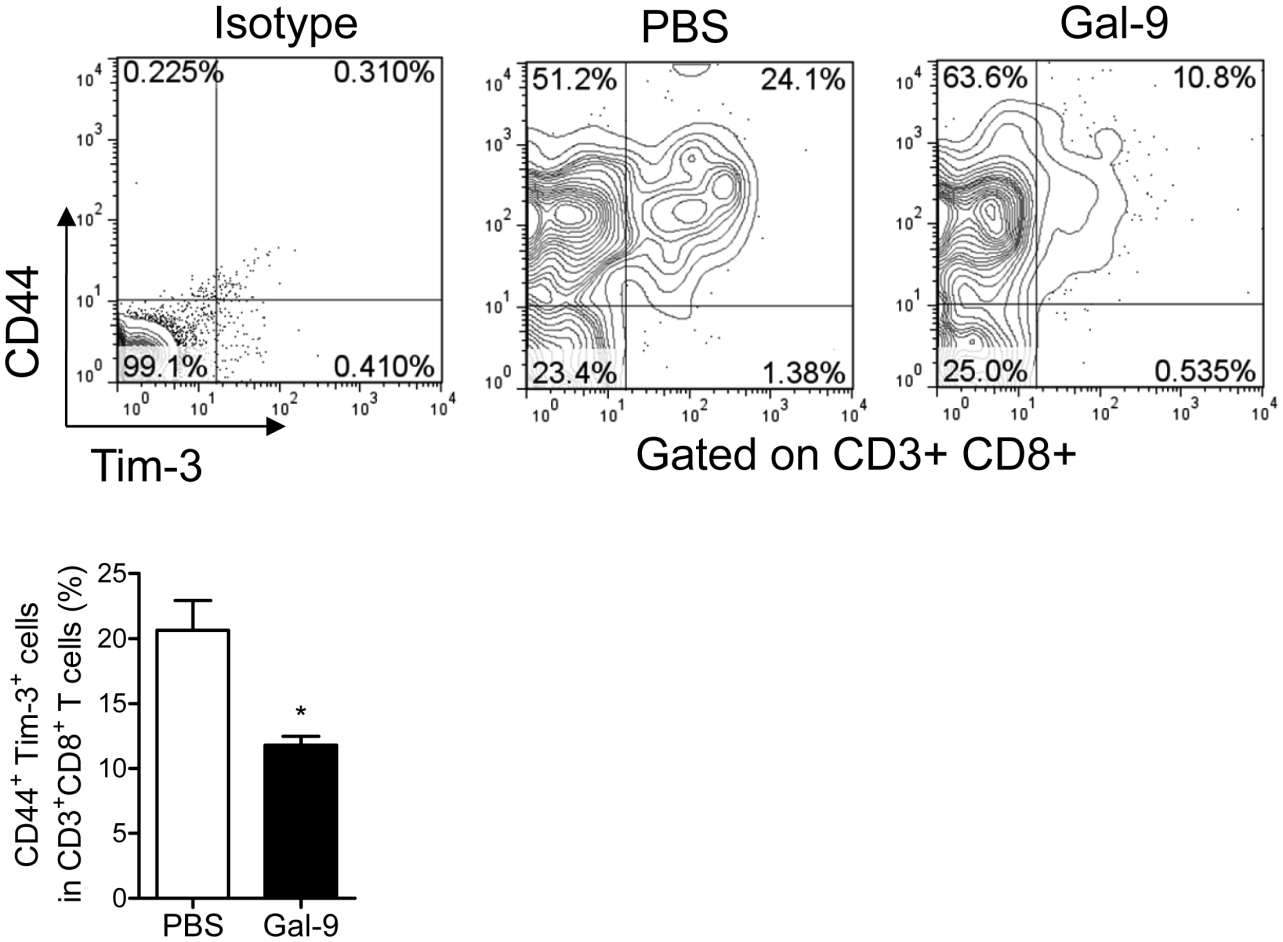
Gal-9 decreases splenic Tim-3^+^ CD44^+^ CD8 T cells. Comparison of the percentage of Tim-3^+^ CD44^+^ CD8^+^ T cells between PBS-treated (n = 4) and Gal-9-treated (n = 4) mice (*, P<0.05). Representative data of flow cytometric profiles are shown.

Moreover, most Tim-3^+^ CD8 T cells in MRL/lpr lupus-prone mice co-expressed CD44, an activated cell marker, and CD44^+^ Tim-3^+^ CD8 T cells decreased significantly in Gal-9-treated mice, suggesting that Gal-9 preferentially decreases Tim-3+ CD8 T cells (**Figure 4**). These results suggest that Gal-9 attenuates disease severity in MRL/lpr lupus-prone mice, at least partly, by regulating Tim-3 expressing effector Th1, Th17, and activated CD8 T cells.

### Gal-9 Suppresses Anti-dsDNA Antibody Production

**Figure 5:**
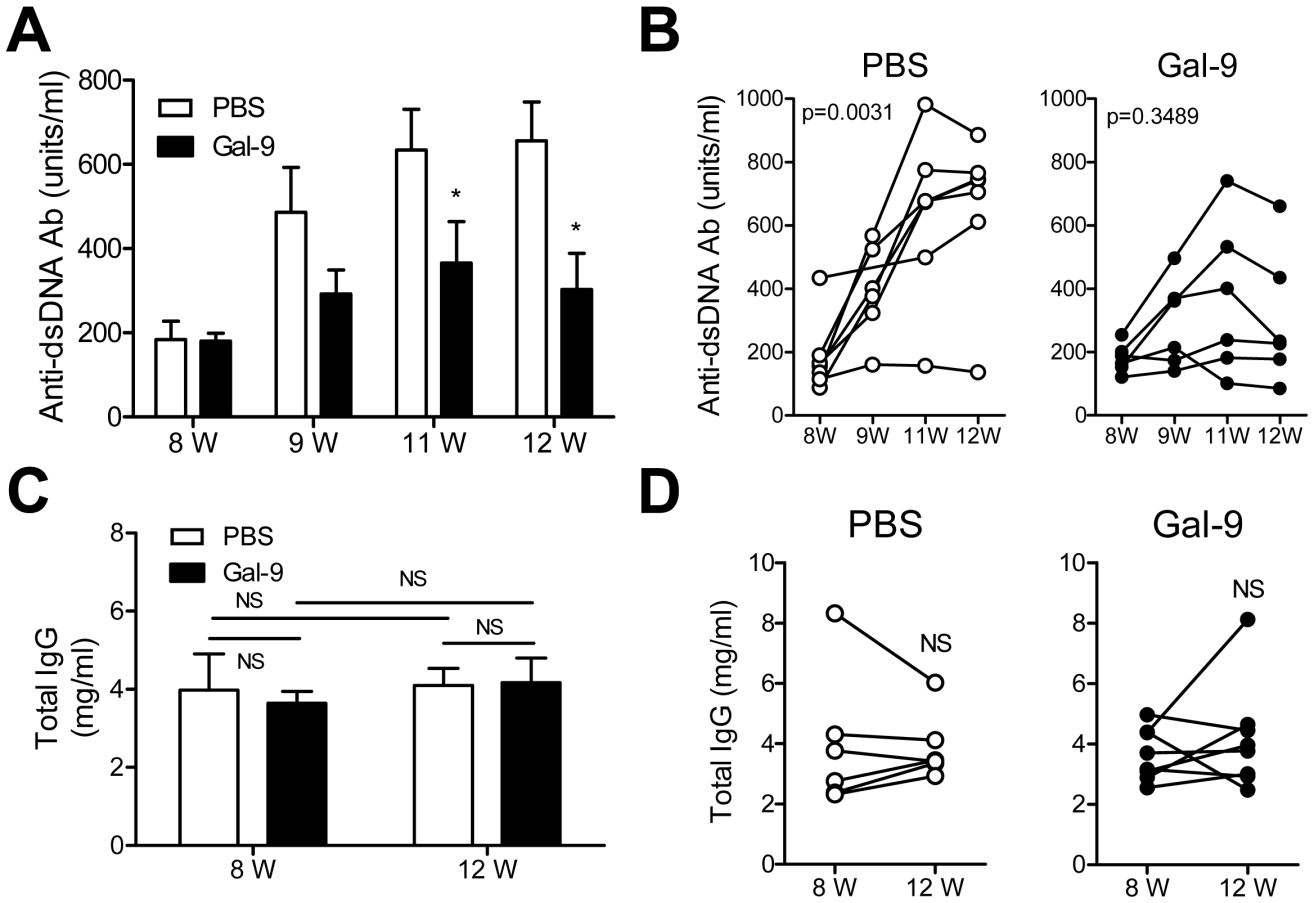
Effects of Gal-9 on the level of total IgG and anti-dsDNA antibody. (**A**) Comparison of anti-dsDNA antibody levels at 8, 9, 11, and 12 weeks of age between PBS-treated (n = 7) and Gal-9-treated (n = 6) mice. Human Gal-9 and PBS were injected intraperitoneally into 8-week-old mice 3-times/week for 4 weeks. (*, P<0.05) (**B**) Comparison of anti-dsDNA antibody levels in 8- to 12-week-old PBS-treated (n = 7) and Gal-9-treated (n = 6) mice. (**C**) Comparison of total IgG levels between PBS-treated (n = 7) and Gal-9-treated (n = 8) mice at 12 weeks of age. Human Gal-9 and PBS were injected intraperitoneally into 8-week-old mice 3-times/week for 4 weeks. (NS, not significant) (**D**) Comparison of total IgG levels in 8- and 12-week-old PBS-treated (n = 7) and Gal-9-treated (n = 8) mice. (NS, not significant).

We hypothesized that Gal-9 suppresses autoantibody production in MRL/lpr lupus-prone mice, because Gal-9 improved hematocrit of MRL/lpr lupus-prone mice. MRL/lpr lupus-prone mice (8-week-old) were treated with Gal-9, since their anti-dsDNA antibody levels began to increase. Gal-9 treatment significantly suppressed anti-dsDNA antibody production in MRL/lpr lupus-prone mice (**Figure 5A**). Moreover, ANOVA analysis confirmed that the level of anti-dsDNA antibody in Gal-9-treated MRL/lpr lupus-prone mice did not increase, whereas the level in PBS-treated mice significantly increased (**Figure 5B**). Next, we performed experiments to ask whether Gal-9 also suppresses the levels of total IgG. In contrast, there was no significant difference in the levels of total IgG between PBS- and Gal-9-treated MRL/lpr lupus-prone mice (**Figure 5C and D**).

### Gal-9 Decreases the Frequency of CD19^−/low^ CD138^+^ Plasma Cells

**Figure 6:**
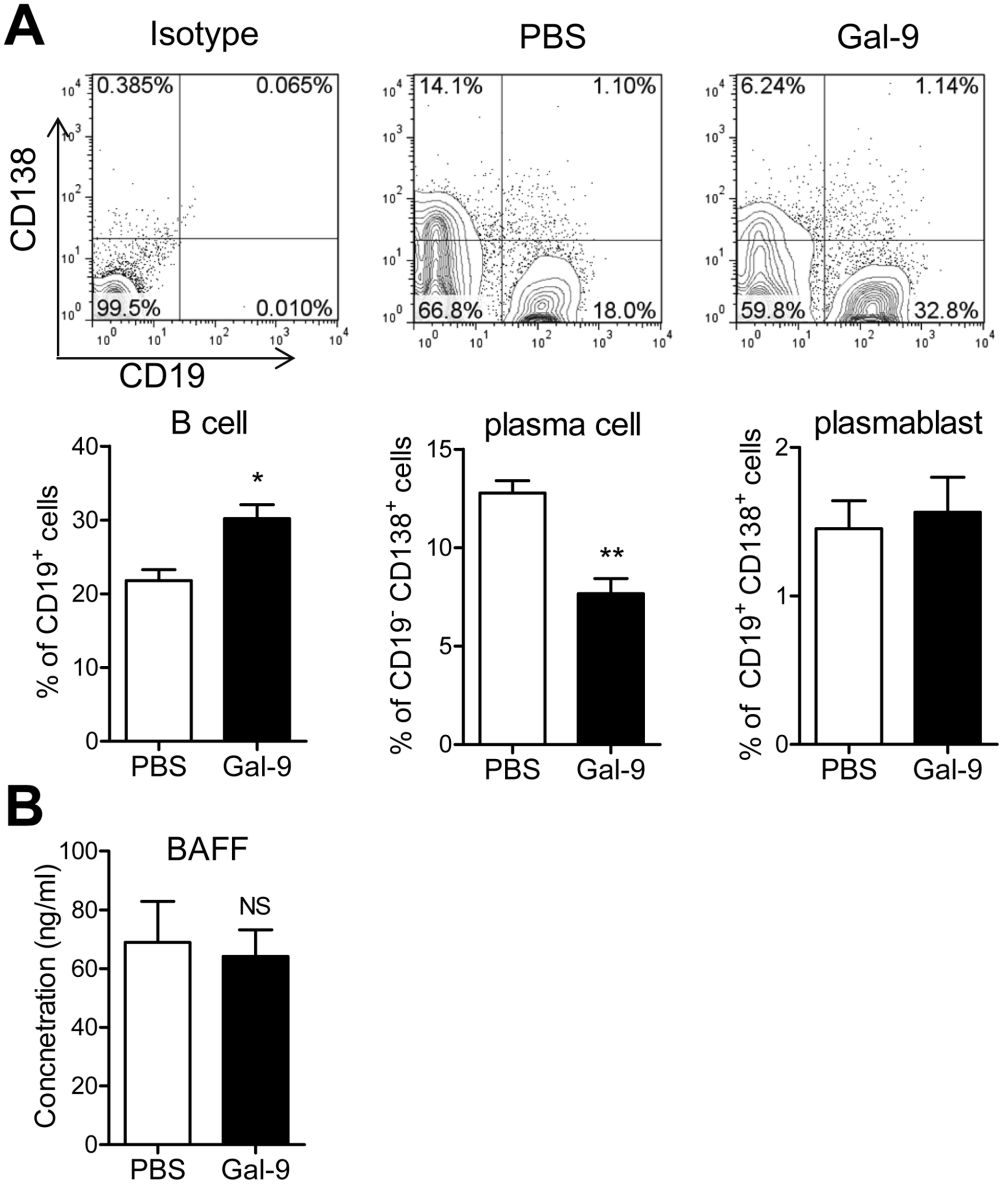
Gal-9 decreases spleen plasma cells in MRL/lpr mice. (**A**) Comparison of the percentage of B cells, plasma cells, and plasmablasts in the spleen of 12-week-old PBS-treated (n = 4) and Gal-9-treated (n = 4) mice. (*, P<0.05, **, P<0.01). Representative data of flow cytometric profiles are shown. (**B**) Comparisons of the level of BAFF between PBS-treated (n = 7) and Gal-9-treated (n = 8) mice at 12-weeks. (NS, not significant).D.

The above results raised the possibility that Gal-9 suppresses anti-dsDNA antibody production through B cell regulation. FACS analysis revealed that the frequency of CD19^+^ B cells was increased in 12-week-old Gal-9-treated MRL/lpr lupus-prone mice (**Figure 6A**). In contrast, Gal-9 significantly reduced the frequency of splenic plasma cells (CD19^−/low^ CD138^+^) but not plasmablasts (CD19^+^ CD138^+^) (**Figure 6A**). It was thus suggested Gal-9 preferentially decreases plasma cells in MRL/lpr lupus-prone mice.

Since BAFF levels are up regulated in SLE patients and MRL/lpr lupus-prone mice to induce plasma cell differentiation [18], [19], [20], [21], we assessed the effects of Gal-9 on serum level of BAFF. ELISA analysis unexpectedly revealed that Gal-9 does not reduce BAFF level (**Figure 6B**). These results suggest that Gal-9 does not decrease plasma cells by downregulating BAFF production, at least in this model.

### Gal-9 Induces Plasma Cell Apoptosis

**Figure 7:**
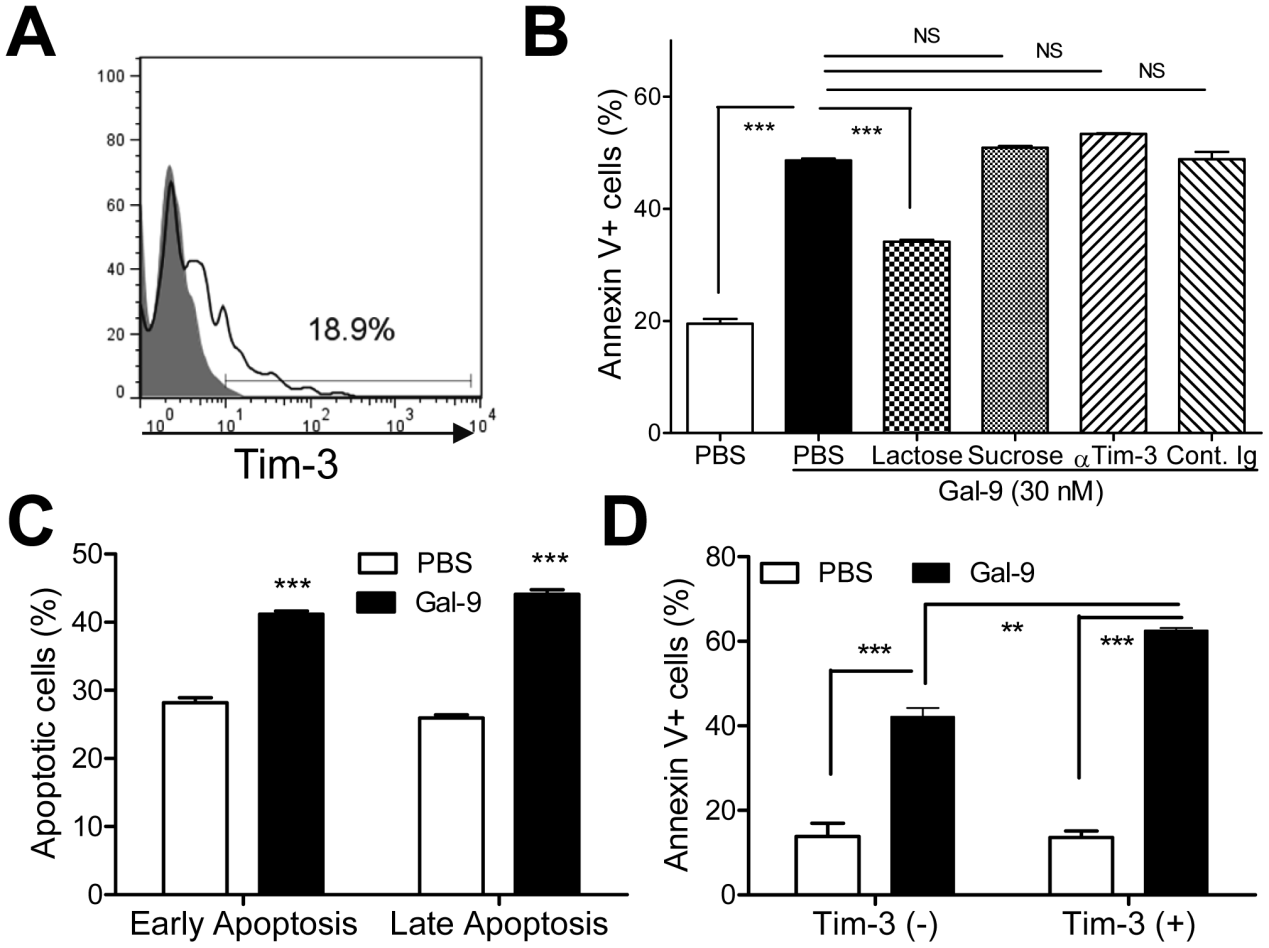
Gal-9 Induces plasma cell apoptosis independently of Tim-3. (**A**) Plasma cells express Tim-3. Tim-3 expression on CD19^−/low^ CD138^+^ cells of spleen cells from 12-week-old MRL/lpr lupus-prone mice was assessed. The results shown are representative data from one of four independent experiments. Filled histogram represents isotype-matched control. (**B**) Induction of plasma cell apoptosis by Gal-9. Plasma cells were treated with 30 nM Gal-9 for 5 h in the presence or absence of lactose, in quadruplicate. (***, P<0.001; NS, not significant). (**C**) Gal-9 induces both early (Annexin V^+^7AAD^−^) and late (Annexin V^+^7AAD^+^) apoptosis of plasma cells from MRL/lpr mice. Plasma cells were treated with 30 nM Gal-9 for 5 h in quadruplicate. (***, P<0.001). (**D**) Susceptibility of Tim-3^+^ plasma cells to Gal-9. Plasma cells were treated with Gal-9 for 5 h. The frequency of Annexin V^+^ cells was assessed in quadruplicate. (***, P<0.001; **, P<0.01; NS, not significant).

Because Gal-9 induces apoptosis of Th1, Th17, and CD8 T cells through Gal-9/Tim-3 interaction, we first asked whether plasma cells express Tim-3, and intriguingly found that about 20% of plasma cells (CD19^−^ CD138^+^) in MRL/lpr lupus-prone mice express Tim-3 (**Figure. 7A**). In order to ask whether Gal-9 induces plasma cell apoptosis, CD19^−^ CD138^+^ cells were prepared by MACS. Gal-9 significantly increased the frequency of Annexin V^+^ apoptotic plasma cells (**Figure 7B**). The apoptosis was significantly suppressed by lactose, whereas anti-Tim-3 antibody unexpectedly did not (**Figure 7B**). Further experiments revealed that Gal-9 induced both early apoptosis (Annexin V^+^7AAD^−^) and late apoptosis (Annexin V^+^7AAD^+^) of plasma cells (**Figure 7C**). Further experiments, however, revealed that Gal-9 induced Tim-3^+^ plasma cell apoptosis than Tim-3^−^ plasma cells (**Figure 7D**). Taken together, Tim-3 may not be directly involved in the apoptosis, and Tim-3 may be associated with vulnerability to Gal-9-mediated plasma cell apoptosis, at least, in MRL/lpr lupus-prone mice.

## Discussion

We found that in MRL/lpr lupus-prone mice, Gal-9 attenuates the severity of various symptoms, such as lupus nephritis, arthritis, and hemolytic anemia. It has been shown that Gal-9 induces apoptosis of Th1 and Th17 cells through Gal-9/Tim-3 interactions [8]. Our recent studies revealed that Gal-9 downregulates Th17 cell differentiation whereas it upregulates differentiation of Foxp3^+^ Tregs, independently of Tim-3 [5]. The beneficial effects of Gal-9 on lupus symptoms in MRL/lpr lupus-prone mice seem partially ascribed to Gal-9-induced decrease of Tim-3^+^ Th1 and Th17 cells because imbalance of Th17 and Th1 cells in SLE and Th17 and Tregs are critical for SLE pathogenesis. Interestingly, Gal-9 failed to expand Foxp3^+^ Tregs in MRL/lpr lupus-prone mice, suggesting that this is attributed to T cell abnormality in MRL/lpr lupus-prone mice [24].

In the present experiments, we also found that CD4^+^ T cells from MRL/lpr lupus-prone mice did not release IL-17 even by PMA stimulation though most of them surprisingly expressed IL-17 in the cytoplasm, suggesting T cell abnormality in the mice. Oppositely, Hou et al recently reported that only less than 1% of cells in CD4 T cells of MRL/lpr mice expressed IL-17 in the cytoplasm [25]. Although heterogeneity in MRL/lpr mice according to the source may be one explanation for the above discrepancy, further studies are, of course, required to ascertain it.

Gal-9 also induces apoptosis of CD8^+^ alloreactive T cells in allografts and viral infections [15], [26], [27], [28]. The fact that Gal-9 also reduces Tim-3^+^ CD44^+^ CD8^+^ T cells in MRL/lpr lupus-prone mice suggests that Tim-3^+^ CD44^+^ CD8^+^ T cells are also associated with lupus pathogenesis in MRL/lpr lupus-prone mice, since infiltrating CD4^+^ and CD8^+^ T cells in lupus kidney indicate that they have the potential to mediate kidney injury [29].

In the present experiments, we show that Gal-9 suppresses anti-dsDNA antibody levels in MRL/lpr lupus-prone mice though the level of total IgG was not changed by Gal-9. Although Gal-9 treatment increased CD19^+^ cells in MRL/lpr lupus-prone mice, it reduced CD19^−^ CD138^+^ plasma cells but not CD19^+^ CD138^+^ plasmablasts. This plasma cell reduction may result in the suppression of anti-dsDNA antibody production.

Recently it was shown that Belimumab, a specific inhibitor of BAFF, and atacicept (TACI-immunoglobulin), a receptor molecule for APRIL, effectively ameliorates clinical symptoms in SLE patients [30], [31], [32]. Although family receptors for BAFF and APRIL vary in their expression patterns and levels across different B-cell subsets, biologic action of BAFF and APRIL may be primarily on memory and/or plasma cells [33], [34]. Moreover, the innate immune system initiates and perpetuates autoimmunity [35]. A small number of patients, undergoing type I IFN therapy for cancer or viral infections, developed SLE. Similarly, IFN-α accelerates SLE in some murine models and is associated with increased BAFF serum levels. Blockade of TNF-α induces increased levels of BAFF via upregulation of type I IFNs and has been associated with development of anti-nuclear antibodies in up to 50% of patients with clinical SLE [37]. B cell depletion in patients treated with anti-CD20 also results in high levels of BAFF, likely an attempt to maintain B cell homeostasis [38].

However, Gal-9 treatment fails to reduce BAFF levels, although it attenuates disease severity. Instead, Gal-9 induces plasma cell apoptosis, suggesting Gal-9 induced plasma cell apoptosis is, at least, partly involved in the suppression of anti-dsDNA antibody production. Of course, It can be raised an alternative explanation that Gal-9 block maturation of CD19^+^ B cells to CD19^−^ plasma cells, because of increased CD19^+^ B cells and decreased plasma cells in MRL/lpr mice. Furthermore, we cannot, however, exclude the possibility of involvement of Gal-9 in the biological function of BAFF. It remains to be clarified whether Gal-9 inhibits binding between BAFF and BAFF receptors, Gal-9 downregulates BAFF receptors on B cells, and suppresses BAFF/BAFF receptor-induced signal transduction.

Furthermore, plasma cells in the spleen of MRL/lpr lupus-prone mice express Tim-3, and the Tim-3^+^ plasma cells are more susceptible to Gal-9-induced apoptosis than the Tim-3^−^ plasma cells. Curiously, a blocking Tim-3 antibody does not abrogate the Gal-9-induced apoptosis. These observations raise several questions. Firstly, as far as we know, this is the first example of Tim-3 expression in the cells of B cell linage. It is, thus, urgently required to ascertain whether Tim-3 expression on plasma cells is limited in MRL/lpr lupus-prone mice or plasma cells in general, including in WT mice and in humans. The second question is whether there are any functional differences between Tim-3^+^ and Tim-3^−^ plasma cells. Tim-3 expression is associated with exhausted phenotypes in virus-infected T-cells and those T-cells are also known to be susceptible to Gal-9-induced apoptosis [40]. The third question is how Tim-3 expression renders plasma cells susceptible to Gal-9, even though Tim-3 may not be the direct target molecule for Gal-9-induced apoptosis. Last, it will be intriguing to clarify whether Gal-9 is also involved in the regulation of B cell development other than plasma cells, including germinal center B cells as they regulate Th17 and Foxp3+ regulatory T cells [7]. Further in-depth studies will be, thus, required to answer the above questions to understand the exact molecular mechanisms of Gal-9-induced regulation of antibody production.

In conclusion, Gal-9 ameliorates clinical severity of MRL/lpr lupus-prone mice by decreasing the level of anti-dsDNA antibody in addition to T cell regulation. The suppressive mechanism was supposed to be the result of induction of apoptosis of plasma cells but not of decrease of BAFF production. Although some CD19^−/low^ CD138^+^ plasma cells in MRL/lpr lupus-prone mice express Tim-3, Gal-9 induced plasma cell apoptosis independently of Tim-3. This suggests that Gal-9 may be a novel candidate for SLE therapy, which would involve an orchestrated mode of action on T cells [41], [42], macrophages [43], [44] and plasma cells.
